# Over-expression of ROR2 and Wnt5a cooperatively correlates with unfavorable prognosis in patients with non-small cell lung cancer

**DOI:** 10.18632/oncotarget.4701

**Published:** 2015-07-21

**Authors:** Chenlin Lu, Xudong Wang, Huijun Zhu, Jian Feng, Songshi Ni, Jianfei Huang

**Affiliations:** ^1^ Department of Respiratory Medicine, Affiliated Hospital of Nantong University, Nantong, Jiangsu, China; ^2^ Department of Laboratory Medicine, Affiliated Hospital of Nantong University, Nantong, Jiangsu, China; ^3^ Department of Pathology, Affiliated Hospital of Nantong University, Nantong, Jiangsu, China

**Keywords:** RTK-like orphan receptor 2 (ROR2), Wnt5a, non-small cell lung cancer, immunohistochemistry, prognosis

## Abstract

We investigated the expression of receptor tyrosine kinase-like orphan receptor (ROR) 2 and Wnt5a and their prognostic significance in non-small cell lung cancer (NSCLC). Tissue microarray-based immunohistochemical analysis was performed to determine the expression of ROR2 and Wnt5a in 219 patients. mRNA expression of *ROR2* and *Wnt5a* was examined in 20 pairs of NSCLC and matched adjacent normal tissues by real-time PCR. Compared with non-tumorous tissues, both mRNA expression and protein product of *ROR2* and *Wnt5a* genes were significantly increased in NSCLC. c^2^ analysis revealed that high ROR2 or Wnt5a expression in NSCLC was significantly associated with advanced TNM stage. High expression of both ROR2 and Wnt5a was also related to advanced TNM stage. Multivariate analyses suggested that ROR2, Wnt5a and TNM stage were independent prognostic factors in NSCLC. Our clinical findings suggest that high ROR2 or Wnt5a expression is associated with poor prognosis in NSCLC, and combined detection of ROR2 and Wnt5a is helpful in predicting the prognosis of NSCLC.

## INTRODUCTION

Lung cancer is one of the primary causes of cancer-related death worldwide, and > 80% of lung cancer patients have non-small cell lung cancer (NSCLC) [1–3]. Despite the improvements in treatment methods (such as surgical resection, chemotherapy, and radiotherapy), the long-term survival of NSCLC is still unsatisfactory, with 5-year survival rate < 10% due to cancer metastasis and relapse [4, 5]. Thus, the identification of useful biomarkers is urgent.

The receptor tyrosine kinase (RTK)-like orphan receptor (ROR)2 belongs to a conserved family of tyrosine kinase receptors that play important roles in some developmental processes, such as chondrogenesis, osteoblastogenesis, and neural differentiation [6, 7]. In recent years, the literature has indicated that ROR2 is implicated in various cancers including metastatic melanoma [8], osteosarcoma [9, 10], and renal cancer [11]. However, expression of ROR2, as well as its prognostic significance has not been evaluated in lung cancer.

ROR2 functions primarily through the Wnt signaling pathway [12]. The Wnt family encodes a large group of signaling molecules that are involved in cell proliferation, differentiation, migration and apoptosis [13, 14]. Wnt signaling can be divided into the canonical and non-canonical pathways [15]. Wnt5a, a typical non-canonical Wnt protein, is related to a variety of malignant tumors [16]. Many previous studies have demonstrated that Wnt5a is upregulated in various cancers, including pancreatic, gastric and prostate cancers [17–19]. By contrast, it sometimes acts as a tumor suppressor in several other cancers such as colon, thyroid and breast cancers [20–23].

Within the Wnt signaling pathway, the main role of ROR2 is to mediate the Wnt5a signals in a complex manner [24, 25]. Recent experiments have confirmed that the Wnt5a-ROR2 signaling cascade is crucial in the aggressive course of osteosarcoma, melanoma, and renal cell carcinoma cell lines [8, 11, 26]. Thus, we investigated the expression of these two markers and assessed their relationship with the clinicopathological features as well as prognosis of NSCLC. We also assessed whether the combined detection of ROR2 and Wnt5a has prognostic value for NSCLC patients.

## RESULTS

### Evaluation of *ROR2* and *Wnt5a* mRNA expression in NSCLC

**Figure 1:**
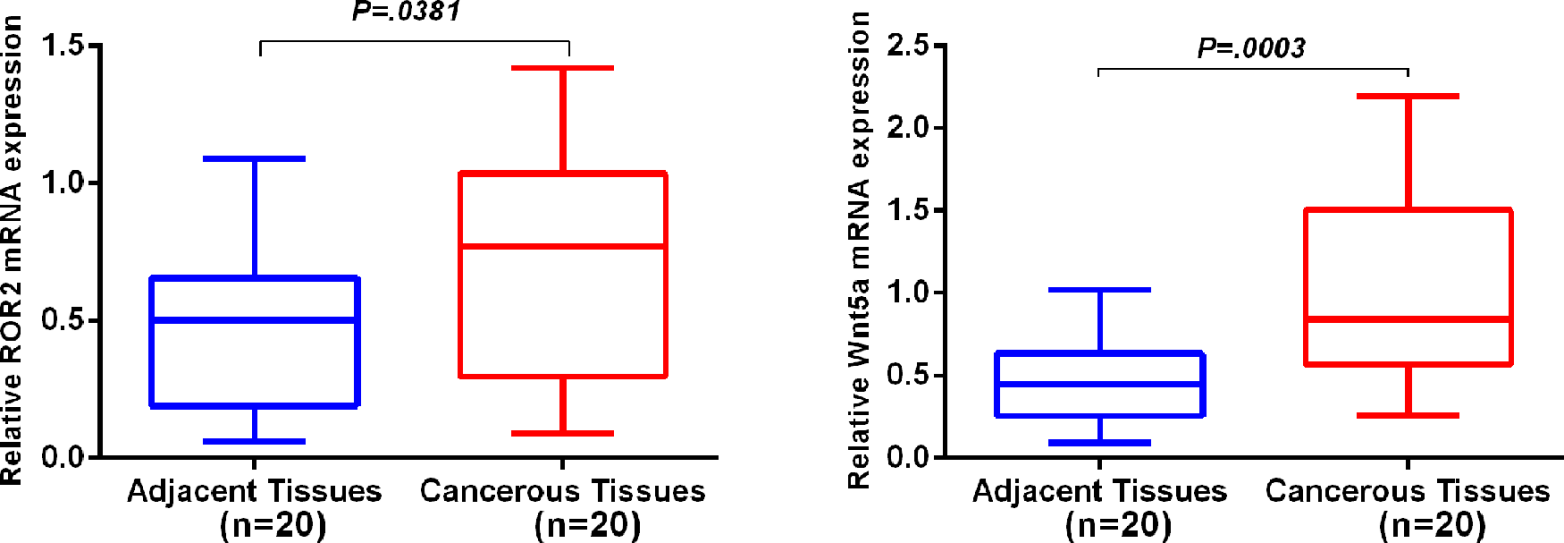
Relative expression of ROR2 and Wnt5a mRNA in NSCLC and adjacent non-cancerous tissues qRT-PCR was performed to elucidate ROR2 and Wnt5a mRNA expression levels in NSCLC compared with tumor-adjacent tissues using 18S rRNA as an endogenous control. ROR2 and Wnt5a mRNA relative expression levels in NSCLC were both significantly higher than those in corresponding non-cancerous tissues (both *P* < 0.05). Error bar is standard error.

Relative expression of *ROR2* and *Wnt5a* mRNA in NSCLC and matched tumor-adjacent tissues were quantified by quantitative reverse transcriptase polymerase chain reaction (qRT-PCR). When normalized to 18S rRNA, *ROR2* mRNA expression level was significantly higher in NSCLC tissues (*n* = 20) compared with matched non-cancerous tissues (*n* = 20) (0.75 ± 0.10 vs 0.5 ± 0.07, *P* = 0.0381), and significantly elevated Wnt5a mRNA expression level in NSCLC was also found (0.99 ± 0.12 vs 0.47 ± 0.06, *P* = 0.0003) (Figure 1).

### Expression of ROR2 and Wnt5a protein in NSCLC detected by immunohistochemistry

**Figure 2:**
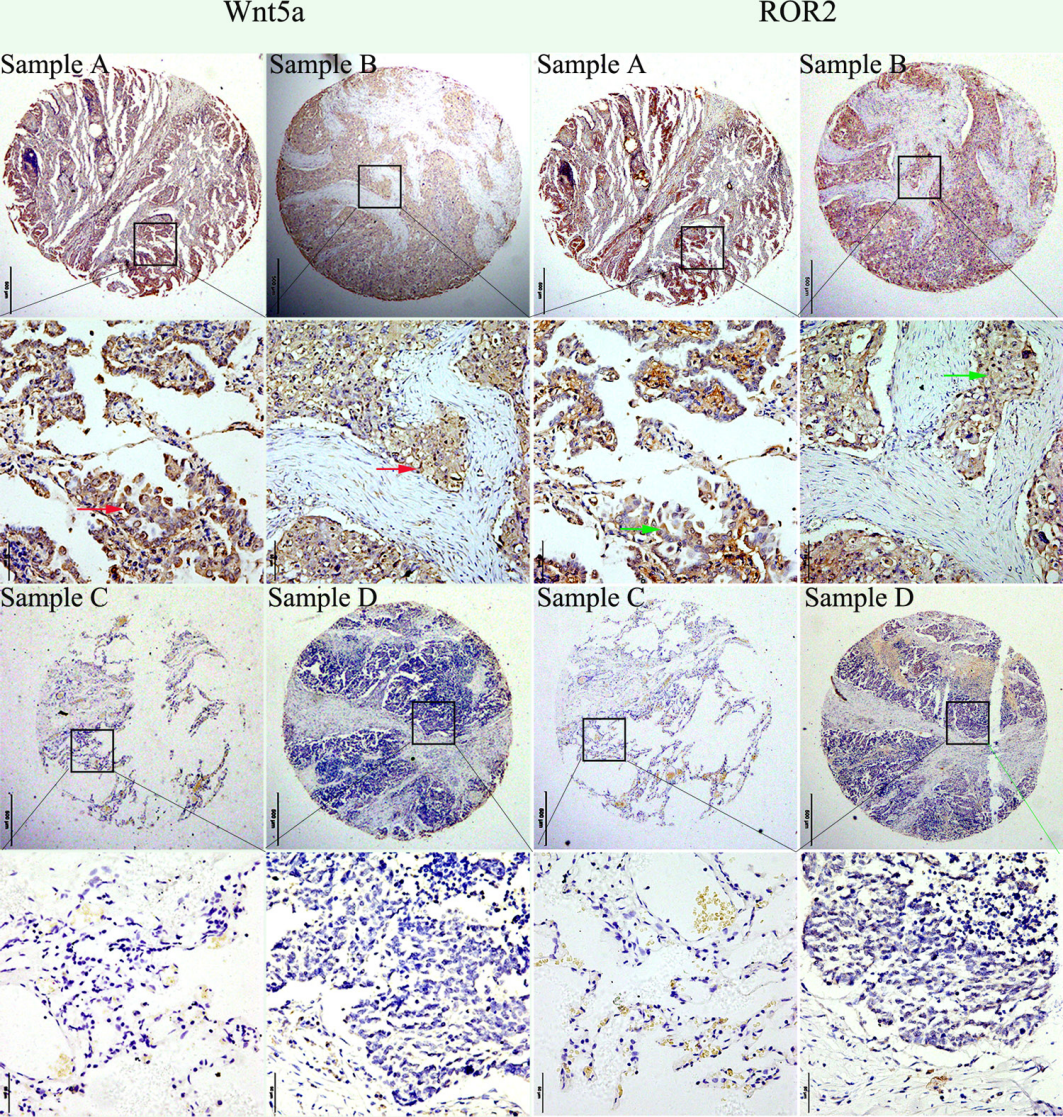
Expression of ROR2 and Wnt5a. Representative photographs of ROR2 and Wnt5a expression in NSCLC and adjacent non-cancerous tissue ROR2 expression was more frequently detected in specimens that stained positively for Wnt5a. **Sample A.** Strong positive immunohistochemical staining (staining intensity score: 3 ) of ROR2 and Wnt5a in NSCLC (adenocarcinoma). **Sample B.** Moderate immunohistochemical staining (staining intensity score: 2) of ROR2 and Wnt5a in NSCLC (squamous cell carcinoma). **Sample C.** Negative immunohistochemical staining (staining intensity score: 0) of ROR2 and Wnt5a in tumor adjacent non-cancerous tissue. **Sample D.** Negative immunohistochemical staining (staining intensity score: 0) of ROR2 and Wnt5a in NSCLC (squamous cell carcinoma). Note: Consecutive sections from the same patient were immunostained with ROR2 and Wnt5a separately. Sample C was the matched adjacent non-cancerous tissue for NSCLC Sample A from the same patient. Red arrows indicated positive WNT5a staining in the membrane and cytoplasm of cells, while green arrows indicated positive ROR2 staining in the cytoplasm and cytoplasmic membrane of cells.

To confirm the results of qRT-PCR, we examined ROR2 and Wnt5a expression in 219 NSCLC tissues and 143 matched adjacent normal lung tissues by immunohistochemistry (IHC). IHC analysis indicated that ROR2 antibody mainly stained the membranes of NSCLC cells; in some cases, the positive ROR2 staining was also observed in cytoplasm (Figure 2), which was in accordance with previous studies [27, 28]. Cells with ROR2 staining in the cytoplasm, cytoplasmic membrane, or both locations were considered as ROR2-positive cells, so did Wnt5a.

For the purpose of statistical analysis, expression intensity of the two makers were dichotomized using X-tile software program-determined cutoff point, with cutoff points for ROR2 and WNT5a being 60 and 90 respectively. Tissues with protein staining score no less than cutoff point were considered “high or positive” expression, and otherwise, would be considered as “low or negative” expression.

High ROR2 expression was found in 49.8% (109/219) of tumors, significantly higher than in 8.39% (12/143) matched, peritumoral tissues (χ^2^ = 66.5685, *P* < 0.001). Similarly, NSCLC samples (35.6%) significantly more frequently showed high Wnt5a expression than matched, adjacent non-tumor tissues (11.19%) (χ^2^ = 26.8527, *P* < 0.001). These findings were consistent with qRT-PCR results. Representative immunohistochemical staining for ROR2 and Wnt5a in NSCLC tissues is shown in Figure 2.

### Correlation of ROR2 and Wnt5a expression with clinicopathological features

To determine the role of ROR2 and Wnt5a in NSCLC progression, we analyzed the relationship between them and major clinicopathological features of NSCLC. ROR2 expression in NSCLC tissues was significantly related to TNM stage (*P* = 0.022) (Table 1). However, it was not related to other clinical features, such as age, sex, pathological type, tumor differentiation, lymph node metastasis (N) and tumor status (T). Wnt5a showed a positive association with TNM stage (*P* = 0.030), but not with other variables (Table 1). Moreover, tumors with high expression of both ROR2 and Wnt5a (ROR2^+^/Wnt5a^+^) were significantly positively related to TNM stage. ROR2 was more frequently detected in specimens that stained positively for Wnt5a (Figure 2), and the Spearman correlation test validated the positive correlation between ROR2 and Wnt5a expression in NSCLC (*r* = 0.347, *P* < 0.001).

**Table 1:** Relationship between expression of ROR2 and Wnt5a tumor tissues and clinicopathological characteristics in NSCLC

Groups	*n*	ROR2 expression			Wnt5a expression			ROR2+/Wnt5a+ expression		
		High(%)	Pearson χ^2^	*P*	High (%)	Pearson χ^2^	*P*	ROR2+/Wnt5a+(%)	Pearson χ^2^	*P*
Total	219	109(49.77)			78(35.62)			57(26.00)		
Age										
≤ 60 years	84	37(44.04)	1.786	0.181	30(35.71)	0.001	0.981	21(25.00)	0.075	0.785
> 60 years	135	72(53.33)			48(35.56)			36(26.67)		
Gender										
Male	167	88(52.69)	2.404	0.121	65(38.92)	3.352	0.067	48(28.74)	2.693	0.101
Female	52	21(40.38)			13(25.00)			9(17.31)		
Differentiation										
Low grade	48	25(52.08)	0.016	0.898	21(43.75)	0.910	0.340	15(31.25)	0.403	0.526
Middle and high grade	147	75(51.02)			53(36.05)			39(26.53)		
Others	24	9			4			3		
Histological type										
Adenocarcinoma	118	66(55.93)	0.127	0.722	47(39.83)	0.286	0.593	35(29.66)	0.280	0.597
Squamous cell carcinoma	38	20(52.63)			17(44.74)			13(34.21)		
Others^a^	63				14			9		
T										
Tis+T1	72	36(50.00)	2.518	0.284	26(36.11)	0.042	0.979	18(25.00)	0.135	0.935
T2	123	59(47.97)			44(35.77)			33(26.83)		
T3+T4	21	14(66.67)			8(38.10)			6(28.57)		
Unknown	3	0			0					
N										
N0	120	54(45.00)	2.581	0.275	37(30.83)	2.720	0.257	26(21.67)	2.874	0.238
N1	54	29(53.70)			23(42.59)			18(33.33)		
N2	45	26(57.78)			18(40.00)			13(28.89)		
TNM stage										
0-I	89	35(39.33)	7.627	0.022*	23(25.84)	7.011	0.030*	14(15.73)	9.287	0.010*
II	72	41(56.94)			32(44.44)			26(36.11)		
III-IV	55	33(60.00)			23(41.82)			17(30.91)		
Unknown	3	0			0					

* *P* < 0.05

a others, Adenosquamous carcinoma and others.

### Overexpression of ROR2 alone, or combined with Wnt5a predicts poorer prognosis

**Figure 3:**
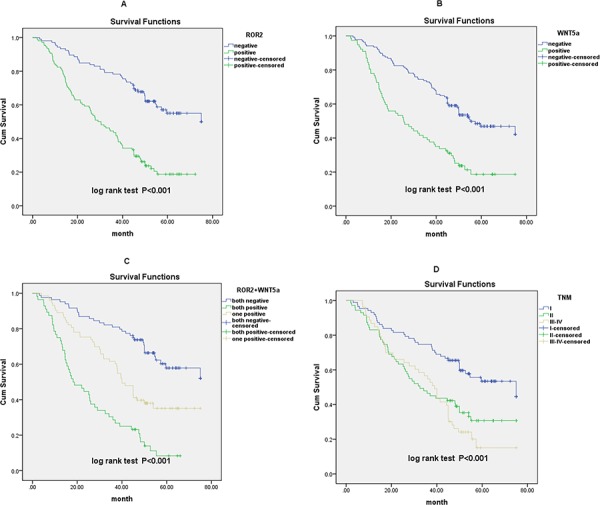
Survival curves for NSCLC patients after surgical therapy **A.** Patients with high ROR2 expression exhibited significantly poorer survival comp ared with the low or no expression group. **B.** Patients with high Wnt5a expression exhibited significantly poorer survival compared with the low or no expression group. **C.** Overall survival rate was significantly lower in patients with both high expression of ROR2 and Wnt5a than that in other groups. **D.** Overall survival rate in patients with advanced TNM stage was significantly lower than that in patients with early TNM stage.

To confirm the role of ROR2 and Wnt5a in NSCLC progression, we used the Cox proportional hazards regression model. We divided the specimens into three groups: group 1, tumors with low expression of two proteins (ROR2^−^/Wnt5a^−^, 89 specimens); group 2, tumors with ROR2^+^/Wnt5a^−^ or ROR2^−^/Wnt5a^+^ (73 specimens); and group 3, tumors with ROR2^+^/Wnt5a^+^ (57 specimens). Based on univariate analysis, high ROR2 expression (*P* < 0.001), high Wnt5a expression (*P* < 0.001), tumor TNM stage (*P* < 0.001), tumor status (T) (*P* = 0.040), lymph node metastasis (N) (*P* < 0.001) and high expression of both ROR2 and Wnt5a (*P* = 0.001) were associated with 5-year survival rate of NSCLC patients. Multivariate analysis further demonstrated that ROR2 expression (*P* < 0.001), Wnt5a expression (*P* = 0.003) and tumor TNM stage (*P* = 0.002) were independent prognostic factors (Table 2). Kaplan-Meier survival curves also revealed that NSCLC patients with high ROR2 expression, high Wnt5a expression, and advanced TNM stage had significantly poorer prognosis (Figure 3). It is notable that patients with the ROR2^+^/Wnt5a^+^ phenotype had an exceptionally poorer prognosis, and showed significantly shorter survival time than patients in the other two groups (Figure 3).

**Table 2:** Univariate and multivariate analysis of different prognostic factors for 5-year survival in patients with NSCLC

Varible	Univariate analysis			Multivariate analysis		
	HR	*P*	95% CI	HR	*P*	95% CI
ROR2 expression	3.113	<0.001*	2.146–4.517	2.716	<0.001*	1.698–4.345
High vs low						
Wnt5a expression	2.500	<0.001*	1.759–3.553	1.774	0.003*	1.221–2.576
High vs low						
ROR2/Wnt5a expression	1.403	0.001*	1.158–1.701	0.924	0.569	0.704–1.213
Both positive vs one positive vs both negative						
Age(years)	1.040	0.830	0.730–1.481			
≤ 60 vs > 60						
Gender	0.931	0.730	0.621–1.396			
Male vs female						
Differentiation	1.015	0.959	0.580–1.775			
Well and moderate vs poor						
Histological type	1.157	0.149	0.949–1.411			
Sq vs Ad vs others						
T	1.361	0.040*	1.015–1.827			
Tis+T1 vs T2 vs T3+T4						
N	1.448	<0.001*	1.183–1.773			
N0 vs N1 vs N2						
TNM stage	1.553	<0.001*	1.256–1.919	1.410	0.002*	1.133–1.755
0-Ivs II vs III-IV						

* *P* < 0.05. TNM stage contains T stage and N stage, therefore, they were not included in the multivariate analysis.

## DISCUSSION

ROR2 is a transmembrane protein that belongs to the ROR family of RTKs. ROR2 exerts its function predominantly via the Wnt signaling pathway [12, 29–31]. For example, Minami first proved that ROR2 mediates Wnt5a-induced activation of the non-canonical pathway [24]. Mikels affirmed that ROR2 mediates inhibition of the β-catenin-dependent Wnt signaling pathway [6]. ROR2 over-expression is also demonstrated to activate c-Jun N-terminal kinase (JNK), a component of the non-canonical Wnt pathway, which has pro-tumorigenic effects [11, 26]. ROR2 has pro-tumorigenic effects in renal cell carcinoma, osteosarcoma and melanoma cell lines in *in vitro* and xenograft experiments [8, 11, 26]. Accumulating evidence indicates that ROR2 may have great potentials for targeted therapy [32, 33]. Many studies have attempted to determine the prognostic values of ROR2 and Wnt5a in various cancers [8, 11, 28, 34–37]. However, the role of ROR2 in the prognosis of NSCLC patients has not been evaluated.

In the present study, we found that the expression of ROR2 and Wnt5a was significantly higher in NSCLC tissues than in matched adjacent normal tissue in both mRNA and protein levels. The high expression of ROR2, Wnt5a, or both proteins was correlated with TNM stage. Univariate analysis showed that cytoplasmic ROR2 expression, Wnt5a expression, tumor TNM stage, tumor status, lymph node metastasis, and combined ROR2 and Wnt5a expression level were correlated with overall survival of NSCLC patients. Mulitivariate analysis indicated that high ROR2 or Wnt5a expression independently predicted poor prognosis of NSCLC. However, it was noteworthy that the significant results for ROR2+/Wnt5a+ versus non-ROR2+/Wnt5a+ in univariate analysis became not significant in multivariable logistic regression analysis. It might suggest that multivariate analysis with the inclusion of multiple dependent variables require larger sample size to detect the significance. Therefore, the non-significant result in the multivariate analysis might be a result of small sample size. Our findings need further validation in the studies with larger sample size. In addition, our data showed that patients with double positive cancers (ROR2+/Wnt5a+) had significantly worse survival than those with double negative cancers (ROR2-/Wnt5a-). The results are similar to previously reported data from studies on various malignancies [10, 17, 27, 38]. Expression of ROR2 and Wnt5a was correlated, supporting the viewpoint that ROR2 acts as a co-receptor for Wnt5a [24, 25]. The correlation was confirmed using Spearman correlation method (*r* = 0.347, *P* < 0.001). Such correlation was also reported in osteosarcoma [36] and hepatocellular carcinoma [28]. However, because the sample size of this study was relatively small, the correlation between ROR2 and Wnt5a in NSCLC are needed to be further verified in studies with large sample size, and *in vitro* and *in vivo* studies should be performed to clarify the underlying mechanism of the correlation.

Accordingly, several studies have provided *in vitro* evidence supporting the role of ROR2 in carcinogenesis [8, 9, 11, 39]. The effect of loss of ROR2 function was evident in renal cell carcinoma (RCC) cells. The loss of ROR2 led to reduced RCC cell migration and anchorage-independent growth as indicated by *in vitro* experiments and an orthotopic xenograft mouse model [11]. Morioka et al. reported that overexpression of ROR2 in human fibroblast and kidney cells conferred increased invasive activity, vice versa [9]. Additionally, metastatic invasion was inhibited in ROR2-silenced melanoma cells in a mouse model [8].

Taken together, these studies suggest that ROR2 exerts oncogenic activity and may be an ideal candidate as a prognostic biomarker and novel therapeutic target for some human cancers. This is believed to be the first study to analyze the role of ROR2 in NSCLC. Recent studies have also indicated that ROR2 has an important role in the Wnt signaling pathway [12]. The Wnt family is a large family of homologous but distinct genes, which have been shown to encode the multifunctional signaling glycoproteins that are involved in the regulation of a variety of physiological and pathological processes, including embryogenesis, differentiation, and tumor formation [40–44]. Wnt5a is one of the important members in the large family, and it is correlated with various cancers [17, 18, 45]. On the one hand, it has a tumor-promoting role in various cancers, including gastric, breast, pancreatic and prostate cancer [17, 19, 46]. On the other hand, it is reported to act as a tumor suppressor gene in several cancers [22, 23]. Several studies have demonstrated that Wnt5a is upregulated in lung cancer, especially in squamous cell carcinoma, and overexpression of Wnt5a is associated with poor prognosis in NSCLC patients [47, 48].

Here, we investigated expression of ROR2 and Wnt5a in NSCLC and assessed their role in prognosis of NSCLC patients. Within the Wnt signaling pathway, ROR2 acts as a receptor or co-receptor for Wnt5a [24, 25]. On the one hand, ROR2 can bind to Wnt5a to activate the non-classical Wnt5a/JNK signaling pathway [24, 49], while on the other hand, ROR2 can also mediate inhibition of the β-catenin-dependent canonical Wnt signaling pathway [6, 30, 31, 50, 51]. The role of ROR2 and Wnt5a in cancer is complex, depending on the tumor type and molecular context. No previous study has examined the concomitant effect of ROR2 and Wnt5a on the prognosis of patients with NSCLC.

Our study also has some limitations: first, this is a retrospective study, the findings might not apply to the general population. Larger prospective studies are needed to confirm our conclusion. Second, we used TMA to assess ROR2 and Wnt5a protein level, the protein expression pattern that we detected might not represent the whole tissue, thus leading to potential biases in the data. Third, the IHC data is semiquantitative, additional methods are needed to evaluate and confirm the expression level of ROR2 and Wnt5a in tumor cells. Fourth, TMN stage is a known independent prognostic factor, with more advanced stage predicting worse survival. However, in the current manuscript, the Kaplan-Meier curves for patients with stage II and III disease did not separate well, which might indicted a bias in the enrollment of the patients in this study. Fifth, because of relative small sample size of the study, we did not explore the effect of target proteins on survival regarding the histology type. Finally, underlying mechanism based on cell lines and other experiment models is needed to support the potential application of both proteins in lung cancer.

In conclusion, expression of ROR2 and Wnt5a is significantly increased in NSCLC tissues and is associated with unfavorable prognosis, indicating that ROR2 and Wnt5a might be used as novel clinicopathological biomarkers to evaluate the prognosis of NSCLC. Our findings also suggest that ROR2 and Wnt5a are promising therapeutic targets and their combined detection is valuable in assessing the prognosis of NSCLC.

## MATERIALS AND METHODS

### Patients and tissue samples

Formalin-fixed, paraffin-embedded NSCLC samples (*n* = 219) and matched, tumor-adjacent specimens (*n* = 143) were collected from 219 patients who underwent surgery at the Affiliated Hospital of Nantong University, Jiangsu, China from 2004 to 2009. At the time of surgery, patients' age ranged from 35 to 83 years, with a median of 62.9 years. No patient received chemotherapy or radiotherapy before the operation. Clinical data were obtained by medical records in the archives room at the hospital. The data included patient sex, age, smoking status, tumor size, tumor differentiation, histological type, tumor status (T), lymph node metastasis (N), distant metastasis (M), and TNM stage. Follow-up data were obtained through telephone investigation. The last follow-up was May 30, 2013. Cancer stage was classified according to the guidelines of the 7^th^ edition of TNM staging in lung cancer [52]. Informed consent has been obtained from all patients before surgery, and the study protocol was approved by the Research Ethics Committee of the Affiliated Hospital of Nantong University.

### qRT-PCR analysis

Fresh frozen NSCLC tissues (*n* = 20) and matched normal, tumor-adjacent tissue samples (*n* = 20) were collected from the Department of Pathology at the Affiliated Hospital of Nantong University for qRT-PCR analysis. Total RNA was extracted using RNeasy Plus Mini Kit (74134; Qiagen, Hilden, Germany), and reverse transcribed to cDNA using High Capacity RNA-to-cDNA Kit (4387406; Life, USA). Real-time RT-PCR was performed using a human ROR2 assay kit (Hs00896176_m1; Life) and Wnt5a assay kit (Hs00998537_m1; Life), as well as TaqMan Universal Master Mix II (4440038; Life) on an ABI7500 system. ROR2 and Wnt5a expression level were normalized against the 18S rRNA (4453320; Life). The experiment was performed in triplicate.

### TMA construction and immunohistochemistry

NSCLC and matched, tumor-adjacent tissues were prepared and used for TMAs. The TMAs were assembled using a tissue arraying instrument (Quick-Ray, UT06; UNITMA, Korea). Core tissue samples (2 mm in diameter) were taken from individual paraffin-embedded sections and deposited in recipient paraffin blocks. TMA specimens were cut into 4-μm sections and put on the super frost-charged glass microscope slides before immunohistochemical processing. Immunohistochemical analysis was performed as previously described. The slides were incubated with the primary antibodies against ROR2 (1:100; LS-C99125, LifeSpan BioSciences, Seattle, WA, USA) or Wnt5a (1:200; Abcam, Cambridge, MA, USA) at 4°C overnight. Horseradish-peroxidase-conjugated rabbit IgG (Abcam) was applied as the secondary antibody for ROR2, and anti-mouse IgG (Abcam) was applied for Wnt5a. The binding of the primary antibody was detected using diaminobenzidine solution. Slide in which primary antibody was omitted was used a negative control, while a breast cancer sample known to be ROR2 positive was included as a positive control.

### Evaluation of immunohistochemistry reaction intensity

Slides were evaluated by two pathologists who were blinded to the patients' prognosis. Expression of ROR2 and Wnt5a was analyzed based on the intensity of staining and the relative number of cells stained. The staining intensity was scored as 0 (no staining), 1 (weak intensity), 2 (moderate intensity), and 3 (strong intensity). The percentage of ROR2- or Wnt5a-positive cells was scored. The product of the intensity and percentage score was used as the final ROR2/Wnt5 staining score, ranging from 0 (no staining) to 300 (100% of cells with 3+ staining intensity). A cutoff point was used to divide tissues into “low or negative” protein expression and “high or positive” protein expression. The cutoff point for the ROR2./Wnt5a expression score that was statistically significant in terms of survival was obtained using the X-tile software program (Rimm Laboratory at Yale University; http://www.tissuearray.org/rimmlab), as previously described [53].

### Statistical analysis

A χ^2^ test was conducted to test the correlation of expression of ROR2 and Wnt5a with clinicopathological variables in the NSCLC group. Spearman correlation test was used to check the correlation between ROR2 and Wnt5a expression in NSCLC. The survival curves were calculated using the Kaplan-Meier method and the log-rank test was used for survival analysis. Factors shown related to prognosis with the univariate Cox regression model were evaluated with the multivariate Cox regression model. Differences were regarded as statistically significant at *P* < 0.05. All statistical analyses were performed using SPSS version 20.0 statistical software (SPSS Inc., Chicago, IL, USA).
